# Efficient electrochemical determination of dopamine in the presence of uric acid in real samples using tungsten disulfide nanostructure modified electrode

**DOI:** 10.5599/admet.2968

**Published:** 2025-10-09

**Authors:** Ibrahim Ayad Jihad, Thekrayat Joodi Jassim, Zainab Naeif Mageed

**Affiliations:** 1 Department of Chemistry and Biochemistry, Al-Zahraa College of Medicine, University of Basrah, Iraq; 2 College of Education/Department of Chemistry/University of Sumer, Thi_Qar, Iraq; 3 Department of Chemistry, College of Science, Mustansiriah University, Baghdad, Iraq

**Keywords:** Central nervous system, cardiovascular system, nanostructured materials, chemically modified electrode

## Abstract

**Background and purpose:**

Research on the detection of uric acid (URA) and dopamine (DPA) is ongoing because of the difficulties posed by their closely overlapping oxidation potentials. Tungsten disulfide nanostructures have become attractive electrode materials to address this problem due to their low toxicity, low cost, easy production, and strong catalytic activity.

**Experimental approach:**

For voltammetric detection of compounds, we present the creation of an electrochemical sensor based on a glassy carbon electrode modified with tungsten disulfide nanostructures.

**Key results:**

According to electrochemical analyses, the manufactured sensor performed exceptionally well, having a broad LDR of 0.03 to 600.0 μM and a low LOD of 10 nM for DPA.

**Conclusion:**

The effective detection of compounds in real samples, such as injections and urine, with acceptable recovery rates further confirmed the suggested sensor's practical usefulness. In addition to offering a viable method for creating tungsten disulfide-based modified electrodes, this study holds promise for future applications in bioanalytical sensing and clinical diagnostics.

## Introduction

**Figure 1.**
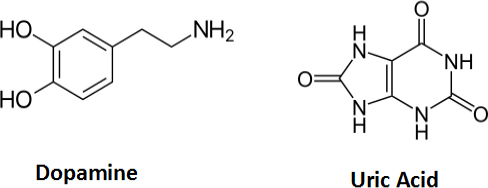
Chemical structure of compounds

Two tiny biomolecules that are essential to human metabolism and other life processes are dopamine (DPA) and uric acid (URA) (Figure 1).

These substances commonly coexist in the serum and the extracellular fluids of the central nervous system (CNS), where they perform vital physiological roles [1]. Due to its vital role in controlling the functioning of the CNS and cardiovascular system, DPA, a major neurotransmitter in the catecholamine family, has garnered considerable interest in medical studies. In clinical settings, DPA is used to treat bacterial infections, heart attacks and in open-heart surgery. Low DPA levels are intimately associated with neurological conditions (Parkinson's disease, schizophrenia, and Alzheimer's disease) [2-4].

The human body contains different amounts of URA, a hydrophilic antioxidant [5]. Notably, the human body does not have an enzyme that can break down URA, and its build-up has been linked to several illnesses, including diabetes [6], gout [7], Lesch-Nyhan syndrome [8], hypercholesterolemia [9] and abnormalities of the kidneys and heart [10]. Therefore, in the fields of neurology, diagnostics, and pharmacological monitoring, it is crucial to develop a rapid and accurate technique for the simultaneous and individual detection of DA and URA. These biomolecules have been detected using a variety of analytical methods, including colorimetry [11,12], fluorescence spectroscopy [13,14] and high-performance liquid chromatography (HPLC) [15,16]. Many of the previously published techniques, however, have a number of drawbacks, including the need for expensive solvents and labor-intensive sample preparation processes. The creation of straightforward, affordable, quick, stable, and extremely sensitive sensors for the simultaneous detection of DA and URA is made possible by electrochemical sensing methods, which provide a more beneficial option [11-16].

The total detection effectiveness of electrochemical sensors is largely dependent on the functioning of the working electrode [17]. One important approach for improving the electrochemical performance of the working electrode is to modify it using materials that are extremely catalytically active [17]. Strategies for modifying electrodes to reduce overpotential, enhance electrocatalytic activity, and improve detection sensitivity have been the subject of extensive research. Additionally, these changes help to minimize electrode surface contamination, reduce undesirable side reactions, and improve stability. The science of electrochemistry has undergone a significant transformation in recent years, largely due to the incorporation of nanotechnology, specifically the functionalization of electrode surfaces with nanomaterials [18].

Nanomaterials' high surface-to-volume ratios enable them to interact effectively with analytes, allowing for the detection of compounds at trace levels with remarkable efficiency [18]. Additionally, their superior electrical conductivity decreases the limit of detection (LOD) and greatly increases sensor sensitivity, resulting in extremely accurate and exact readings. This development demonstrates how nanoparticles can revolutionize contemporary electrochemical sensing applications. Due to their exceptional mechanical stability, large surface area, and electrical conductivities, two-dimensional transition metal dichalcogenides (TMDCs) have become a potential material for electrode modification [19].

A plane of metal atoms is encased between two hexagonally organized chalcogenide layers in layered TMDCs, which have an X–M–X architecture. Disulfides of transition metals have garnered a lot of interest because of their high energy storage capacity, graphene-like layered structure, and high electrical conductivity, similar to tungsten disulfide (WS_2_) [20]. Because of its unique layered structure, high capacitance, and superior inherent electrical conductivity, tungsten disulfide is a promising material for electrochemical electrodes. WS_2_ is regarded as one of the best transition metal dichalcogenides for use as an electrode and in energy storage applications. This is primarily due to its enormous specific surface area, which stems from its S-W-S layered structure, which is covalently bonded in two dimensions [21-23].

Furthermore, tungsten atoms in tungsten disulfide may reside in a variety of oxidation states (+2 to +6), allowing for flexible redox activity. Additionally, WS_2_ may store charge via the intercalation process, making it a promising pseudocapacitive material for cutting-edge energy storage devices [21-23].

In the present study, we utilized a WS_2_/GCE for the simultaneous detection of URA and DA. The WS_2_/GCE demonstrated outstanding performance for DA detection, as demonstrated by voltammetry experiments. It is distinguished by a low LOD and a broad LDR. Additionally, the sensor's capacity to identify DA and URA was carefully examined. The manufactured sensor's outstanding analytical performance and practical application were demonstrated.

## Experimental

### Reagents and instruments

DA, URA and other reagents were prepared from commercial suppliers. The Micro AUTOLAB potentiostat-galvanostat (Metrohm, the Netherlands) was subjected to chronoamperometry tests and voltammetric techniques to examine the electrochemical investigations and measurements. Additionally, the electrochemical cell utilized in this study had three electrode systems: the reference electrode was Ag/AgCl (KCl 3.0 M), the counter electrode was Pt wire, and the working electrode (WE) was either an unmodified GCE or a modified GCE.

### Synthesis of WS_2_ nanostructures

With minor adjustments, a hydrothermal technique based on a method published in the literature was used to create WS_2_ nanostructures [24]. To do this, 30 mL of deionized water was used to dissolve 2.2 mmol of WCl_6_ and 22 mmol of thioacetamide, which were then agitated for 15 minutes at room temperature. For 40 minutes, it was ultrasonically treated. After that, the prepared solution was placed in an autoclave lined with Teflon and subjected to hydrothermal treatment for 24 hours at 240 °C.

The autoclave was removed and cooled at room temperature once the hydrothermal treatment was finished. To remove the pollutants, the prepared sediments were collected, sorted, and repeatedly washed with deionized water and ethanol. Ultimately, the WS_2_ nanostructures were produced following a 15-hour vacuum oven drying process at 70 °C.

### Preparation of the sensor

0.5 mL of deionized water was mixed with 0.5 mg of the WS_2_ nanostructures, and the mixture was homogenized for 20 minutes using ultrasonics. WS_2_/GCE was then formed by drop-casting 4.0 μL of the well-dispersed suspension onto the GCE surface and allowing it to cure at room temperatures.

## Results and discussion

### Characterization of WS_2_ nanostructures

**Figure 2.**
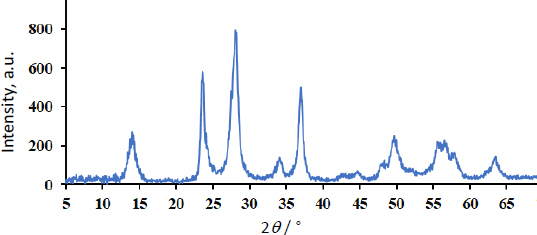
XRD pattern of WS_2_ nanostructures

The XRD pattern, shown in Figure 2, confirmed the generated sample's crystal structure and phase purity.

Diffractions of the (002), (004), (100), (101), (103), (006), (105), (110), (008), (112) and (107) planes of WS_2_ are responsible for the peaks at 14.0, 28.1, 34.0, 36.9, 39.7, 44.6, 49.6, 55.6, 56.5, 57.7 and 63.3°, respectively [25,26]. On the other hand, the diffraction peak at 23.6° may be associated with WO_3_ impurities [27].

### Electrochemical behaviour of DPA at unmodified GCE and WS_2_/GCE

**Figure 3.**
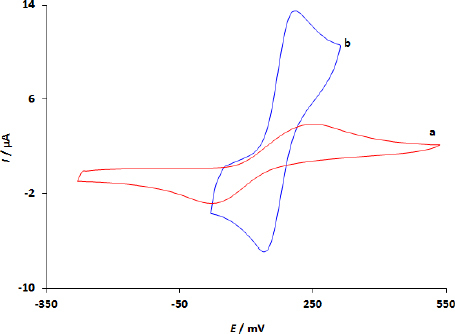
CVs of 100.0 μM DPA on the unmodified GCE (a) and WS_2_/GCE (b).

Cyclic voltammograms of DPA (100.0 μM) at the unmodified GCE and WS_2_/GCE electrodes are shown in Figure 3. The voltammograms consist of one current peak pair. This indicated that DPA under these circumstances underwent a redox electrochemical process. The unaltered GCE showed a sluggish electrochemical response associated with the DPA redox process (Figure 3a), while the voltammogram obtained at modified WS_2_/GCE demonstrates that the WS_2_ nanostructures facilitate the charge transfer reaction, causing increasing currents and lower potentials.

### Effects of scan rate

**Figure 4.**
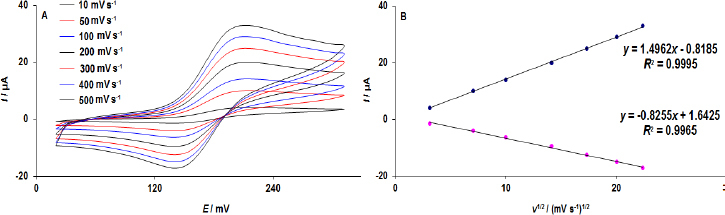
(A) CV responses of WS_2_/GCE in DPA (75.0 μM) at different scan rates; (B) corresponding relationship of *I_pa_* and *I_pc_* vs. *ν*^1/2^

Using the CV method, the effect of scan rate on the redox reaction of DA at WS_2_/GCE was determined at a concentration of 75.0 μM at various scan rates (Figure 4A). According to the observed CVs, the DPA redox peak currents increase with increasing scan rates. Furthermore, the currents of DPA showed linear dependencies on *ν*^1/2^, as shown in Figure 4B. This demonstrates the diffusion-controlled nature of the DPA redox process at the WS_2_/GCE.

### Chronoamperometric investigations

**Figure 5.**
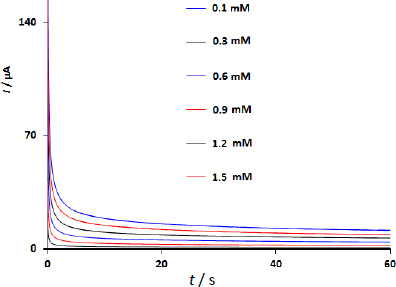
Chronoamperograms of WS_2_/GCE in DA at varying doses

Using chronoamperometry studies at 0.25 V for 60 s in DA at increasing concentrations from 0.1 to 1.5 mM, the diffusion coefficient of DPA at the WS_2_/GCE was determined (Figure 5).

The chronoamperograms show that the currents increase with an increase in DA concentration. The Cottrell equation may be used to get the diffusion coefficient (*D*):



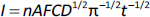

(1)


**Figure 6.**
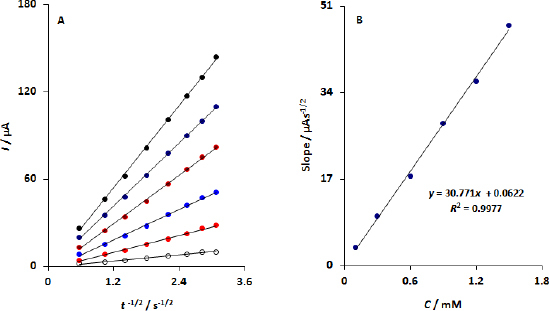
(A) Corresponding plot of *I* vs. t^-1/2^ at certain periods from figure 5 are shown in Inset A; (B) A corresponding plot of the slopes derived from *I vs. t*^-1/2^ plots

Plots of anodic peak current versus *t*^-1/2^ were plotted (Figure 6A). The slopes increase linearly with concentration of DA (Figure 6B). Based on slope and Cotrell equation, the DPA diffusion coefficient at the modified CPE was 8.08×10^-5^ cm^2^ s^-1^.

### Electroanalytical determination of DPA at WS_2_/GCE

**Figure 7.**
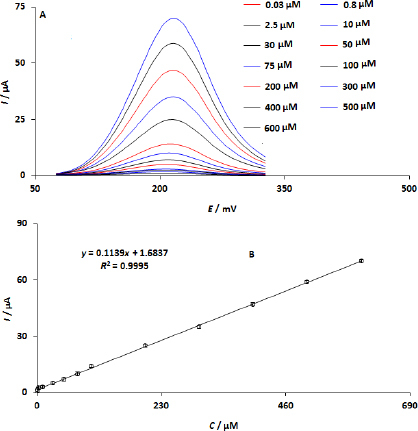
(A) DPA DPV responses of WS_2_/GCE with different concentrations of DPA; (B) corresponding calibration curve of *I* against DPA concentrations

The electroanalytical determination of DA was carried out using DPV measurements at varying concentrations (Figure 7A). The current responses increase with increasing DPA concentration. Additionally, a linear correlation was found between DPA concentrations (ranging from 0.03 to 600.0 μM) and the recorded current responses. Figure 7B shows the DPA calibration curve. Moreover, the LOD was determined to be 1.0 nM (*S*/*N* = 3).

### Electrochemical performance of WS_2_/GCE for simultaneous determination

**Figure 8.**
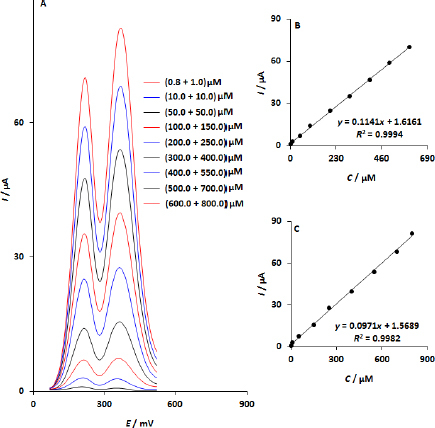
(A) WS_2_/GCE DPV responses with varying doses of DPA and URA. *I*_pa_ *vs.* DPA (B) and URA (C) calibration graphs that correspond to each other

The DPV responses of WS_2_/GCE in a solution of URA and DPA at different concentrations are shown in Figure 8A. At 210 mV and 360 mV, two distinct peaks were seen.

As expected, the currents of both species increase with increasing concentration (Figures 8B and 8C). The results indicate that WS_2_/GCE may be effectively used to simultaneously determine these species.

### Analysis of urine and DPA samples

The concentration of DPA and URA in urine and DPA injection samples was examined. First, the amounts of URA and DPA in the prepared samples were determined, and the DPV responses of WS_2_/GCE were recorded from unspiked samples.

This study was then carried out by increasing the known concentrations of DPA and URA in the samples. The recoveries were calculated using the conventional addition process. Table 1 illustrates the high accuracy of the designed sensing platform, with good recovery values (ranging from 97.1 to 104.5 % for DPA and 97.1 to 104.0 % for URA).

**Table 1.** The obtained results for the analysis of URA and DPA in the urine samples and DPA injection.

Sample	Concentration, M				Recovery, %		RSD, % (*n* = 5)	
	Spiked		Measured					
DPA Injection	DPA	URA	DPA	URA	DPA	URA	DPA	URA
	0	0	4.4	-	-	-	3.6	-
	1.5	5.0	6.0	4.9	101.7	98.0	2.1	3.1
	2.5	7.5	6.7	7.6	97.1	101.3	3.0	2.7
	3.5	10.0	7.8	10.4	98.7	104.0	1.9	2.1
	4.5	12.5	9.3	12.4	104.5	99.2	2.7	2.5
Urine	0	0	-	2.4	-	-	-	2.7
	5.0	2.0	5.1	4.3	102.0	97.7	3.0	2.3
	7.0	4.0	6.8	6.5	97.1	101.6	2.2	3.3
	9.0	6.0	9.1	8.2	101.1	97.6	2.7	1.8
	11.0	8.0	10.9	10.8	99.1	103.8	2.3	2.5

## Conclusions

In conclusion, a voltammetric sensor was constructed using the WS_2_/GCE to simultaneously detect URA and DPA. The electro-catalytic activity of the voltammetric sensor was excellent for detection. The DPA detection capabilities of this WS_2_/GCE included a low limit of detection (10 nM), strong selectivity, acceptable repeatability, a broader linear range (0.03 to 600.0 μM), and better sensitivity. Furthermore, two distinct oxidation signals were acquired for the coexistence of DPA and URA. Additionally, the examination of physiological residues and pharmaceutical samples has shown promising findings.
